# Second Order Duality in Multiobjective Fractional Programming with Square Root Term under Generalized Univex Function

**DOI:** 10.1155/2014/541524

**Published:** 2014-07-07

**Authors:** Arun Kumar Tripathy

**Affiliations:** Department of Mathematics, Trident Academy of Technology, F2/A, Chandaka Industrial Estate, Bhubaneswar, Odisha 751024, India

## Abstract

Three approaches of second order mixed type duality are introduced for a nondifferentiable multiobjective fractional programming problem in which the numerator and denominator of objective function contain square root of positive semidefinite quadratic form. Also, the necessary and sufficient conditions of efficient solution for fractional programming are established and a parameterization technique is used to establish duality results under generalized second order *ρ*-univexity assumption.

## 1. Introduction

A fractional programming problem arises in many types of optimization problems such as portfolio selection, production, information theory, and numerous decision making problems in management science. More specifically, it can be used in engineering and economics to minimize a ratio of physical or economical function or both, such as cost/time, cost/volume, and cost/benefit, in order to measure the efficiency or productivity of the system. Many economic, noneconomic, and indirect applications of fractional programming problem have also been given by Bector [1], Bector and Chandra [2], Craven [3], Mond and Weir [4], Stancu-Minasian [5], Schaible and Ibaraki [6], Ahmad et al. [7], Ahmad and Sharma [8], and Gulati et al. [9].

The central concept in optimization is known as the duality theory which asserts that, given a (primal) minimization problem, the infimum value of the primal problem cannot be smaller than the supermom value of the associated (dual) maximization problem and the optimal values of primal and dual problems are equal. Duality in fractional programming is an important class of duality theory and several contributions have been made in the past [1, 5, 8, 10–14]. Second order duality provides a tighter bound for the value of the objective function when approximations are used. For more details, one can consult [15, page 93]. Another advantage of second order duality when applicable is that if a feasible point in the primal is given and first order duality does not apply, then we can use second order duality to provide a lower bound of the value of the primal problem (see [4]).

Multiobjective fractional programming duality has been of much interest in the recent past. Schaible [16] and Bector et al. [11] derived Fritz John and Karush-Kuhn Tucker necessary and sufficient optimality condition for a class of nondifferentiable convex multiobjective fractional programming problems and established some duality theorems. Liang et al. [17, 18] discussed the optimality condition and duality for nonlinear fractional programming. Santos et al. [19] discussed the optimality and duality for nonsmooth multiobjective fractional programming with generalized convexity. Bector et al. [20] and Xu [21] gave a mixed type duality for fractional programming, established some sufficient conditions, and obtained various duality results between the mixed dual and primal problem. Zhou and Wang [22] introduced a class of mixed type dual for nonsmooth multiobjective fractional programming and established the duality results under (*V*, *ρ*) invexity assumption.

Duality for various forms of mathematical problems involving square roots of positive semidefinite quadratic forms has been discussed by many authors [10, 23–25]. Mond [25] considered a nonlinear fractional programming problem involving square roots of positive semidefinite quadratic form in the numerator and denominator and proved the necessary and sufficient condition for optimality. Kim et al. [26, 27] formulated a nondifferentiable multiobjective fractional problem in which numerators contain support function. One of the most known approaches used for solving nonlinear fractional programming problem is called parametric approach. Dinklebaeh [28] and Jagannathan [12] introduced this approach that was used later by Osuna-G$\overset{´}{\text{o}}$mez et al. [13] to characterize solution of a multiobjective fractional problem under generalized convexity. Tripathy [14] introduced three approaches given by Dinklebaeh [28] and Jagannathan [12] for both primal and mixed type dual of a nondifferentiable multiobjective fractional programming and established the duality results under generalized *ρ*-univexity.

To relax convexity assumption imposed on the function in theorems on optimality and duality, various generalized convexity concepts have been proposed. Hanson [29] introduced the class of invex functions. Bector et al. [30] introduced univex function. Mishra [31] derived the optimality condition for multiobjective programming with generalized univexity. Jayswal [32] presented minimax fractional programming under generalized *ρ*-univexity assumption.

Motivated by the earlier authors in this paper, we have introduced three approaches given by Dinklebaeh [28] and Jagannathan [12] for both primal and second order mixed type dual of a nondifferentiable multiobjective fractional programming problem in which the numerator and denominator of objective function contain square root of positive semidefinite quadratic form. Also we have established the necessary and sufficient optimality condition and used a parameterization technique to establish duality results under generalized *ρ*-univexity assumption.

## 2. Notations and Preliminaries

Let *ℝ* ^*n*^ be the *n*-dimensional Euclidean space and *ℝ*_+_^*n*^ its nonnegative orthant. The following conventions for inequality will be used throughout this paper. For any *x* = (*x*_1_, *x*_2_,…, *x*_*n*_), *y* = (*y*_1_, *y*_2_,…, *y*_*n*_), we denote the following.

(i) *x* > *y*⇔*x*_*i*_ > *y*_*i*_, for all *i* = 1,2,…, *n*.
(ii) *x* ≥ *y*⇔*x*_*i*_ ≥ *y*_*i*_, for all *i* = 1,2,…, *n*.

Throughout the paper, let *X* be a nonempty open subset of *ℝ* ^*n*^.

Consider the following nondifferentiable multiobjective fractional programming problem.

### 2.1. Multiobjective Fractional Primal Problem

(i) * MFP0*. Minimize
  (1) $$\begin{array}{c} \frac{f\left(x\right)+{\left(x^{T}Bx\right)}^{1/2}}{g\left(x\right)-{\left(x^{T}Cx\right)}^{1/2}}=\left(K_{1}\left(x\right),K_{2}\left(x\right),\ldots ,K_{k}\left(x\right)\right), \end{array}$$
 where
  (2) $$\begin{array}{c} K_{i}\left(x\right)=\frac{f_{i}\left(x\right)+{\left(x^{T}B_{i}x\right)}^{1/2}}{g_{i}\left(x\right)-{\left(x^{T}C_{i}x\right)}^{1/2}},\;i=1,2,\ldots ,k. \end{array}$$
(ii) * MFP1*. Minimize
  (3) $$\begin{array}{c} F\left(x\right)=\left(F_{1}\left(x\right),F_{2}\left(x\right),\ldots ,F_{k}\left(x\right)\right), \end{array}$$
 where
  (4) Fi(x)=fi(x)+(xTBix)1/2−νi{gi(x)−(xTCix)1/2},hhhhhhhhhhhhhhhhhhhhhhhhhhhhhhhi=1,2,…,k;
 
*ν*_*i*_ are fixed parameters.
(iii) *MFPλ*. Minimize *λF*(*x*); *λ* is *k*-dimensional strictly positive vector,
 all subject to same constraint

(5) $$\begin{array}{c} h\left(x\right)\leq 0,\;x\in X\subseteq R^{n}, \end{array}$$

where *f*_*i*_ : *ℝ* ^*n*^ → *ℝ*, *g*_*i*_ : *ℝ* ^*n*^ → *ℝ*, *i* = 1,2,…, *k* and *h* = (*h*_1_,…, *h*_*m*_); *h*_*j*_ : *ℝ* ^*n*^ → *ℝ*, *j* = 1,2,…, *m*, are differentiable functions, *B*_*i*_ and *C*_*i*_, *i* = 1,2,…, *k* are positive semidefinite matrices of order *n*. In the sequel, we assume that *f*_*i*_(*x*) ≥ 0 and *g*_*i*_(*x*) > 0 on *ℝ* ^*n*^ for *i* = 1,2,…, *k*.

Let *X*_0_ = {*x* ∈ *X*⊆*ℝ* ^*n*^ : *h*_*j*_(*x*) ≤ 0, *j* = 1,2,…, *m*} for all feasible solutions of MFP0, MFP1, and MFP*λ* and denote *I* = {1,2, 3,…, *k*}, *M* = {1,2, 3,…, *m*}, *J*_1_ = {*j* ∈ *M* : *h*_*j*_(*x*) = 0}, and *J*_2_ = {*j* ∈ *M* : *h*_*j*_(*x*) < 0}. It is obvious that *J*_1_ ∪ *J*_2_ = *M*.

Throughout the paper, consider *f*_*i*_ : *X* → *ℝ*, *η* : *X* × *X* → *ℝ* ^*n*^, *p* ∈ *ℝ* ^*n*^, *ρ* ∈ *ℝ*.

Assume that *ψ* : *ℝ* → *ℝ* satisfying *a* ≤ 0⇒*ψ*(*a*) ≤ 0 or *ψ*(*a*) ≤ 0⇒*a* ≤ 0 and *ψ*(−*a*) = −*ψ*(*a*), *K* : *X* × *X* → *ℝ*_+_. For $x,\overset{-}{x}\in X$ we can write $K\left(x,\overset{-}{x}\right)=\lim_{\lambda \to 0}b\left(x,\overset{-}{x},\lambda \right)\geq 0$.

**Definition 1 .**

The real differentiable function *f*_*i*_ is said to be second order *ρ*-univex at $\overset{-}{x}\in X$ with respect to *η*, *ψ*, and *K*, if

(6) K(x,x−)ψ[fi(x)−fi(x−)+12pT(∇2fi(x−)p)] ≥η(x,x−)T[∇fi(x−)+∇2fi(x−)p]+ρ||x−x−||2,ssssssssssssssssssssssssssssssssssssssssssss∀x∈X.

**Definition 2 .**

The real differentiable function *f*_*i*_ is said to be second order *ρ*-pseudounivex at $\overset{-}{x}\in X$ with respect to *η*, *ψ*, and *K*, if

(7) η(x,x−)T[∇fi(x−)+∇2fi(x−)p]+ρ||x−x−||2≥0 ⟹K(x,x−)ψ[fi(x)−fi(x−)+12pT(∇2fi(x−)p)]≥0,sssssssssssssssssssssssssssssssssssssssssssssssssssssss∀x∈X.

**Definition 3 .**

The real differentiable function *f*_*i*_ is said to be second order *ρ*-quasiunivex at $\overset{-}{x}\in X$ with respect to *η*, *ψ*, and *K*, if

(8) K(x,x−)ψ[fi(x)−fi(x−)+12pT(∇2fi(x−)p)]≤0 ⟹η(x,x−)T[∇fi(x−)+∇2fi(x−)p]+ρ||x−x−||2≤0,sssssssssssssssssssssssssssssssssssssssssssssssssss∀x∈X.

**Remark 4 .**

If *p* = 0, the above definitions reduce to the definitions of *ρ*-univex, *ρ*-pseudounivex, and *ρ*-quasiunivex as introduced in [14].

**Definition 5 .**

A feasible point $\overset{-}{x}$ is said to be efficient for MFP1, if there exists no other feasible point *x* in MFP1 such that $F_{i}\left(x\right)\leq F_{i}\left(\overset{-}{x}\right)$, *i* = 1,2,…, *k*, and $F_{r}\left(x\right)<F_{r}\left(\overset{-}{x}\right)$ for some *r* ∈ {1,2,…, *k*}.

**Definition 6 (see [33]).**

A feasible point $\overset{-}{x}$ is said to be properly efficient for MFP1, if it is efficient and there exist *M* > 0 such that, for each *i* ∈ {1,2,…, *k*} and for all feasible point *x* in MFP1 satisfying $F_{i}\left(x\right)<F_{i}\left(\overset{-}{x}\right)$, we have $F_{i}(\overset{-}{x})-F_{i}(x)\leq M(F_{r}(x)-F_{r}(\overset{-}{x}))$ for some *r* ∈ {1,2,…, *k*} such that $F_{r}(x)>F_{r}(\overset{-}{x})$.

We assume that *f*_*i*_(*x*)+(*x* ^*T*^ *B*_*i*_ *x*)^1/2^ ≥ 0, *g*_*i*_(*x*)−(*x* ^*T*^ *C*_*i*_ *x*)^1/2^ > 0, *i* = 1,2,…, *k* for all *x* ∈ *X*.

**Definition 7 (generalized Schwarz Inequality).**

Let *B* be a positive semidefinite matrix of order *n*. Then, for all *x*, *w* ∈ *ℝ* ^*n*^, *x* ^*T*^ *Bw* ≤ (*x* ^*T*^ *Bx*)^1/2^(*w* ^*T*^ *Bw*)^1/2^.

The equality holds if *Bx* = *λBw* for some *λ* ≥ 0.

Let *J*_1_(*x*) = {*j* ∈ *M* = {1,2,…, *m*} : *h*_*j*_(*x*) = 0} and ${\overset{-}{v}}_{i}=\left(f_{i}\left(\overset{-}{x}\right)+{\left(x^{T}B_{i}x\right)}^{1/2}\right)/\left(g_{i}\left(\overset{-}{x}\right)-{\left(x^{T}C_{i}x\right)}^{1/2}\right)$.

Then define the set $W\left(\overset{-}{x}\right)=\left\{w\in {\mathbb{R}}^{n}:w^{T}\nabla h_{j}\left(\overset{-}{x}\right)\leq 0,j\in J\left(\overset{-}{x}\right)\right\}$ satisfying any one of the following conditions:

(a) ${\overset{-}{x}}^{T}B_{i}\overset{-}{x}>0$, ${\overset{-}{x}}^{T}C_{i}\overset{-}{x}=0\Rightarrow w^{T}\left(\nabla f_{i}\left(\overset{-}{x}\right)+B_{i}\overset{-}{x}/({\overset{-}{x}}^{T}B_{i}\overset{-}{x}{)}^{1/2}-{\overset{-}{v}}_{i}\nabla g_{i}\left(\overset{-}{x}\right)\right)+{\left(w^{T}\left({\overset{-}{v}}_{i}^{2}C_{i}\right)w\right)}^{1/2}\geq 0$, $w\in W\left(\overset{-}{x}\right)$, *i* = 1,2,…, *k*;
(b) ${\overset{-}{x}}^{T}B_{i}\overset{-}{x}=0$, ${\overset{-}{x}}^{T}C_{i}\overset{-}{x}>0\Rightarrow w^{T}\left(\nabla f_{i}\left(\overset{-}{x}\right)-{\overset{-}{v}}_{i}\left\{\nabla g_{i}\left(\overset{-}{x}\right)-C_{i}\overset{-}{x}/{\left({\overset{-}{x}}^{T}C_{i}\overset{-}{x}\right)}^{1/2}\right\}\right)+{\left(w^{T}B_{i}w\right)}^{1/2}\geq 0$, $w\in W\left(\overset{-}{x}\right)$, *i* = 1,2,…, *k*;
(c) ${\overset{-}{x}}^{T}B_{i}\overset{-}{x}=0$, ${\overset{-}{x}}^{T}C_{i}\overset{-}{x}=0\Rightarrow w^{T}\left(\nabla f_{i}\left(\overset{-}{x}\right)-{\overset{-}{v}}_{i}\nabla g_{i}\left(\overset{-}{x}\right)\right)+{\left(w^{T}B_{i}w\right)}^{1/2}+{\left(w^{T}\left({\overset{-}{v}}_{i}^{2}C_{i}\right)w\right)}^{1/2}\geq 0$, $w\in W\left(\overset{-}{x}\right)$, *i* = 1,2,…, *k*;
(d) ${\overset{-}{x}}^{T}B_{i}\overset{-}{x}>0$, ${\overset{-}{x}}^{T}C_{i}\overset{-}{x}>0$ $\Rightarrow w^{T}\left(\nabla f_{i}\left(\overset{-}{x}\right)+B_{i}\overset{-}{x}/{\left({\overset{-}{x}}^{T}B_{i}\overset{-}{x}\right)}^{1/2}-{\overset{-}{v}}_{i}\left\{\nabla g_{i}(\overset{-}{x})-C_{i}\overset{-}{x}/{\left({\overset{-}{x}}^{T}C_{i}\overset{-}{x}\right)}^{1/2}\right\}\right)\geq 0$, $w\in W\left(\overset{-}{x}\right)$, *i* = 1,2,…, *k*.

**Lemma 8 (see [33]).**

If *x* ^0^ ∈ *X*_0_ is an optimal solution of *MFPλ*, then *x* ^0^ is properly efficient for MFP1.

**Lemma 9 (see [12]).**

*x* ^0^ ∈ *X*_0_ is an efficient solution for MFP0 if and only if it is an efficient solution of MFP1 with *F*(*x* ^0^) = 0.

**Lemma 10 (see [10] necessary optimality condition).**

If $\overset{-}{x}\in X_{0}$ is an optimal solution of (*MFPλ*) such that $W(\overset{-}{x})=\phi$, then there exist *v*_*i*_ ∈ *ℝ*_+_, *w*, *z* ∈ *ℝ* ^*n*^, and *y* ∈ *ℝ* ^*m*^ such that

(9) ∇λF(x−)+yT∇h(x−) =∑i=1kλi[∇fi(x−)+Biw−vi{∇gi(x−)−Ciz}]  +∑j=1myj∇hj(x−)=0,

(10) Fi(x−)=fi(x−)+(x−TBix−)1/2−vi(gi(x−)−(x−TCix−)1/2)=0,hhhhhhhhhhhhhhhhhhhhhhhhhhhhhhhhhhi=1,2,…,k.

(11) $$\begin{array}{c} y^{T}h\left(\overset{-}{x}\right)=0, \end{array}$$

(12) $$\begin{array}{c} w^{T}B_{i}w\leq 1,\;z^{T}C_{i}z\leq 1,\;i=1,2,\ldots ,k, \end{array}$$

(13) (x−TBix−)1/2=x−TBiw, (x−TCix−)1/2=x−TCiz,hhhhhhhhhhhhhhhhhhhhhhhhi=1,2,…,k,

(14) $$\begin{array}{c} y\geq 0, \end{array}$$

(15) $$\begin{array}{c} v_{i}\geq 0,\;i=1,2,\ldots ,k. \end{array}$$

**Theorem 11 (sufficient optimality condition).**

Let $\overset{-}{x}\in X_{0}$ be a feasible solution of MFP1 and there exist *λ*_*i*_ ∈ *ℝ*_+_; *w*, *z* ∈ *ℝ* ^*n*^, *v*_*i*_ ∈ *ℝ*_+_, and *y* ∈ *ℝ* ^*m*^ satisfying the condition in Lemma 10 at $\overset{-}{x}$. Furthermore suppose that the following conditions hold.

(i) *P*(*x*) = ∑_*i*=1_^*k*^ *λ*_*i*_[*f*_*i*_(*x*) + (*x* ^*T*^ *B*_*i*_ *x*)^1/2^ − *v*_*i*_{*g*_*i*_(*x*) − (*x* ^*T*^ *C*_*i*_ *x*)^1/2^}] + ∑_*j*=1_^*m*^ *y*_*j*_ *h*_*j*_(*x*) is second order *ρ*-pseudounivex with respect to *η*,  *ψ*, and *K* at $\overset{-}{x}\in X_{0}$, with (∇^2^ *P*(*x*))*p* = 0, where *f*_*i*_ : *X* → *ℝ*, *g*_*i*_ : *X* → *ℝ*, *h*_*j*_ : *X* → *ℝ*, *i* = 1,2,…, *k*; *j* = 1,2,…, *m*, *p* ∈ *ℝ* ^*n*^, *η* : *X* × *X* → *ℝ* ^*n*^, *K* : *X* × *X* → *ℝ*_+_, and *ψ* : *ℝ* → *ℝ* satisfying *ψ*(*a*) ≤ 0⇒*a* ≤ 0.
(ii) *ρ* ≥ 0.

Then $\overset{-}{x}$ is an efficient solution of MFP1.

**Proof**

Suppose that the hypothesis holds.

Since the conditions of Lemma 10 are satisfied, from (9) and (13), we have

(16) ∇P(x−) =∇(∑j=1myjhj(x−)∑i=1kλi[fi(x−)+(x−TBix−)1/2ssssssssssss−vi{gi(x−)−(x−TCix−)1/2}]+∑j=1myjhj(x−)) =∇(∑j=1myjhj(x−)∑i=1kλi[fi(x−)+x−TBiw−vi{gi(x−)−x−TCiz}]sssssssssss+∑j=1myjhj(x−))=0.

Also from hypothesis (i), we have $\nabla^{2}P\left(\overset{-}{x}\right)p=0$

So we can write $\nabla P\left(\overset{-}{x}\right)+\nabla^{2}P\left(\overset{-}{x}\right)p=0$.

Now for $\eta \left(x,\overset{-}{x}\right)\in {\mathbb{R}}^{n}$, we can write $\eta {\left(x,\overset{-}{x}\right)}^{T}\left(\nabla P\left(\overset{-}{x}\right)+\nabla^{2}P\left(\overset{-}{x}\right)p\right)=0$.

For *ρ* ≥ 0, we have $\eta {\left(x,\overset{-}{x}\right)}^{T}\left(\nabla P\left(\overset{-}{x}\right)+\nabla^{2}P\left(\overset{-}{x}\right)p\right)+\rho {||x-\overset{-}{x}||}^{2}\geq 0$.

Since *P*(*x*) is second order *ρ*-pseudounivex with respect to *η*,  *ψ*, and *K* at $\overset{-}{x}\in X_{0}$, we have $K\left(x,\overset{-}{x}\right)\psi \left\{P\left(x\right)-P\left(\overset{-}{x}\right)+\left(1/2\right)p^{T}\left(\nabla^{2}P\left(\overset{-}{x}\right)\right)p\right\}\geq 0$, and using the properties of *K*,  *ψ*, it gives

(17) P(x)−P(x−)+12pT∇2P(x−)p ≥0⟹P(x)≥P(x−)−12pT∇2P(x−)p.

Since $\nabla^{2}P\left(\overset{-}{x}\right)p=0$, the above inequality implies $P\left(x\right)\geq P\left(\overset{-}{x}\right)$

(18) ⟹∑i=1kλi[fi(x)+(xTBix)1/2sssssssssss−vi{gi(x)−(xTCix)1/2}]+∑j=1myjhj(x)≥∑i=1kλi[fi(x−)+(x−TBix−)1/2sssssssssss−vi{gi(x−)−(x−TCix−)1/2}]+∑j=1myjhj(x−).

Suppose that $\overset{-}{x}$ is not efficient solution of MFP1; then there exist $\overset{-}{x}\in X_{0}$ such that

(19) fi(x)+(xTBix)1/2−vi{gi(x)−(xTCix)1/2} ≤fi(x−)+(x−TBix−)1/2−vi{gi(x−)−(xTCix)1/2},ssssssssssssssssssssssssssssssssssssssssi=1,2,…,k,

and $f_{t}\left(x\right)+{\left(x^{T}B_{t}x\right)}^{1/2}-v_{t}\{g_{t}\left(x\right)-{\left(x^{T}C_{t}x\right)}^{1/2}\}\leq f_{t}\left(\overset{-}{x}\right)+{\left({\overset{-}{x}}^{T}B_{t}\overset{-}{x}\right)}^{1/2}-v_{t}\left\{g_{t}\left(\overset{-}{x}\right)-{\left(x^{T}C_{t}x\right)}^{1/2}\right\}$, for some *t* ∈ {1,2,…, *k*}.

The above relation, together with the relation *λ*_*i*_ > 0, implies that

(20) ∑i=1kλi[fi(x)+(xTBix)1/2−vi{gi(x)−(xTCix)1/2}] <∑i=1kλi[fi(x−)+(x−TBix−)1/2−vi{gi(x−)−(xTCix)1/2}].

From the relations (5), (11), and (14), we get

(21) $$\begin{array}{c} \overset{m}{\underset{j=1}{\sum }}y_{j}h_{j}\left(x\right)\leq \overset{m}{\underset{j=1}{\sum }}y_{j}h_{j}\left(\overset{-}{x}\right). \end{array}$$

Consequently (20) and (21) yield

(22) ∑i=1kλi[fi(x)+(xTBix)1/2−vi{gi(x)−(xTCix)1/2}]  +∑j=1myjhj(x) <∑i=1kλi[fi(x−)+(x−TBix−)1/2−vi{gi(x−)−(xTCix)1/2}]  +∑j=1myjhj(x−).

This contradicts (18). Hence $\overset{-}{x}$ is an efficient solution for MFP1.

**Theorem 12 (sufficient optimality condition).**

Let $\overset{-}{x}\in X_{0}$ be a feasible solution of MFP1 and there exist *λ*_*i*_ ∈ *ℝ*_+_; *w*, *z* ∈ *ℝ* ^*n*^, *v*_*i*_ ∈ *ℝ*_+_, and *y* ∈ *ℝ* ^*m*^ satisfying the condition in Lemma 10 at $\overset{-}{x}$. Furthermore suppose that the following conditions hold.

(i) *Q*(*x*) = ∑_*i*=1_^*k*^ *λ*_*i*_[*f*_*i*_(*x*) + (*x* ^*T*^ *B*_*i*_ *x*)^1/2^ − *v*_*i*_{*g*_*i*_(*x*) − (*x* ^*T*^ *C*_*i*_ *x*)^1/2^}] is second order *ρ*-pseudounivex with respect to *η*, *ψ*_0_, and *K* at $\overset{-}{x}\in X_{0}$ and *H*(*x*) = ∑_*j*=1_^*m*^ *y*_*j*_ *h*_*j*_(*x*) is second order *σ*-quasiunivex with respect to *η*, *ψ*_1_, and *K* at $\overset{-}{x}\in X_{0}$ with $\left(\nabla^{2}Q\left(\overset{-}{x}\right)\right)p=0$ and $\left(\nabla^{2}H\left(\overset{-}{x}\right)\right)p=0$ where *f*_*i*_ : *X* → *ℝ*, *g*_*i*_ : *X* → *ℝ*, *h*_*j*_ : *X* → *ℝ*, *i* = 1,2,…, *k*; *j* = 1,2,…, *m*, *p* ∈ *ℝ* ^*n*^, *η* : *X* × *X* → *ℝ* ^*n*^, *K* : *X* × *X* → *ℝ*_+_, and *ψ*_0_, *ψ*_1_ : *ℝ* → *ℝ* satisfying *ψ*_0_(*a*) ≥ 0⇒*a* ≥ 0 and *ψ*_1_(*a*) ≤ 0⇒*a* ≤ 0.
(ii) *ρ* + *σ* ≥ 0.

Then $\overset{-}{x}$ is an efficient solution of *MFP*1.

**Proof**

Suppose hypothesis holds.

From the relations (5), (11), and (14), we get

(23) ∑j=1myjhj(x) ≤∑j=1myjhj(x−)⟹H(x)≤H(x−)⟹H(x)−H(x−)≤0.

Also from hypothesis (i), we get $\left(\nabla^{2}H\left(\overset{-}{x}\right)\right)p=0$.

So we have the following:

(24) H(x)−H(x−)+12pT(∇2H(x−))p≤0 ⟹K(x,x−)ψ1{H(x)−H(x−)+12pT(∇2H(x−))p}≤0.

Hence, the *σ*-quasiunivexity of *H*(*x*) with respect to *η*,  *ψ*_1_, and *K* implies the following:

(25) η(x,x−)T{∇H(x−)+(∇2H(x−))p}+σ||x−x−||2≤0 ⟹η(x,x−)T{∇H(x−)}+σ||x−x−||2≤0.

From (9), we get

(26) ∑i=1kλi[∇fi(x−)+Biw−vi{∇gi(x−)−Ciz}]  +∑i=1myi∇hi(x−)=0 ⟹∇Q(x−)+∇H(x−)=0 ⟹η(x,x−)T[∇Q(x−)+∇H(x−)]=0 ⟹η(x,x−)T∇Q(x−)+η(x,x−)T∇H(x−)   +σ||x−x−||2−σ||x−x−||2=0.

Using (25) in (26), we get

(27) $$\begin{array}{c} \eta {\left(x,\overset{-}{x}\right)}^{T}\nabla Q\left(\overset{-}{x}\right)-\sigma {||x-\overset{-}{x}||}^{2}\geq 0. \end{array}$$

Since *ρ* + *σ* ≥ 0, we get

(28) $$\begin{array}{c} \rho {||x-\overset{-}{x}||}^{2}\geq -\sigma {||x-\overset{-}{x}||}^{2}. \end{array}$$

So, we have

(29) η(x,x−)T∇Q(x−)+ρ||x−x−||2≥0 ⟹η(x,x−)T{∇Q(x−)+∇2Q(x−)p}+ρ||x−x−||2≥0.

Since *Q*(*x*) is *ρ*-pseudounivex with respect to *η*,  *ψ*_0_, and *K*, we obtained

(30) $$\begin{array}{c} K\left(x,\overset{-}{x}\right)\psi_{0}\left[Q\left(x\right)-Q\left(\overset{-}{x}\right)+\frac{1}{2}p^{T}\nabla^{2}Q\left(\overset{-}{x}\right)p\right]\geq 0. \end{array}$$

Using the property of *K* and *ψ*_0_, we get

(31) Q(x)−Q(x−)+12pT∇2Q(x−)p≥0⟹Q(x)≥Q(x−) ⟹∑i=1kλi[fi(x)+(xTBix)1/2ssssssssssssss−vi{gi(x)−(xTCix)1/2}] ≥∑i=1kλi[fi(x−)+(x−TBix−)1/2−vi{gi(x−)−(x−TCix−)1/2}].

If $\overset{-}{x}$ were not an efficient solution to MFP1, then, from (20), we have

(32) ∑i=1kλi[fi(x)+(xTBix)1/2−vi{gi(x)−(xTCix)1/2}] <∑i=1kλi[fi(x−)+(x−TBix−)1/2sssssssssss−vi{gi(x−)−(x−TCix−)1/2}].

This contradicts (31).

Therefore, $\overset{-}{x}$ is an efficient solution for MFP1.

## 3. Second Order Mixed Type Multiobjective Fractional Duality

(i) * MMFD0*. Maximize
  (33) L(u) =(L1(u)−12pT∇2L1(u)p, L2(u)  sss−12pT∇2L2(u)p,…,Lk(u)−12pT∇2Lk(u)p),
 where
  (34) $$\begin{array}{c} L_{i}\left(u\right)=\frac{f_{i}\left(u\right)+y_{J_{1}}^{T}h_{J_{1}}\left(u\right)+u^{T}B_{i}w}{g_{i}\left(u\right)-u^{T}C_{i}z},\;i=1,2,\ldots ,k. \end{array}$$
(ii) * MMFD1*. Maximize
  (35) G(u) =(G1(u)−12pT∇2G1(u)p, G2(u)  sss−12pT∇2G2(u)p,…,Gk(u)−12pT∇2Gk(u)p),
 where *G*_*i*_(*u*) = *f*_*i*_(*u*) + *y*_*J*_1__^*T*^ *h*_*J*_1__(*u*) + *u* ^*T*^ *B*_*i*_ *w* − *ν*_*i*_{*g*_*i*_(*u*) − *u* ^*T*^ *C*_*i*_ *z*}, *i* = 1,2,…, *k*; *ν*_*i*_ are fixed parameters.
(iii) *MMFDλ*. Maximize *λG*(*u*); *λ* is *k*-dimensional strictly positive vector,
 all subject to same constraints

(36) ∑i=1kλi[∇Gi(u)+∇2Gi(u)p]+yJ2T[∇hJ2(u)+∇2∇hJ2(u)p] =0,

(37) fi(u)+yJ1ThJ1(u)+uTBiw−vi{gi(u)−uTCiz}≥0,hhhhhhhhhhhhhhhhhhhhhhhhhhfor  i=1,2,…,k.

(38) yJ2ThJ2(u)−12pT∇2(yJ2ThJ2(u))p≥0,hhhhhhhhhhhhhhhhhhyJ2∈Rm−|J1|,

(39) $$\begin{array}{c} w^{T}B_{i}w\leq 1,\;z^{T}C_{i}z\leq 1,\;i=1,2,\ldots ,k, \end{array}$$

(40) $$\begin{array}{c} y\geq 0,\;v_{i}\geq 0,\;\;i=1,2,\ldots ,k, \end{array}$$

where *f*_*i*_ : *X* → *ℝ*, *g*_*i*_ : *X* → *ℝ*, *h*_*j*_ : *X* → *ℝ*, *i* = 1,2,…, *k*; *j* = 1,2,…, *m* are differentiable functions, *w*, *z* ∈ *ℝ* ^*n*^, *p* ∈ *ℝ* ^*n*^. *B*_*i*_, and *C*_*i*_, *i* = 1,2,…, *k* are positive semidefinite matrices of order *n*.

For the following theorems, we assume that *η* : *X* × *X* → *ℝ* ^*n*^, *K* : *X* × *X* → *ℝ*_+_, and *ψ*_0_, *ψ*_1_ : *ℝ* → *ℝ* satisfying *ψ*_0_(*a*) ≥ 0⇒*a* ≥ 0 and *b* ≤ 0⇒*ψ*_1_(*b*) ≤ 0 and *ρ*, *σ* ∈ *ℝ*.

**Theorem 13 (weak duality).**

Let *x* be a feasible solution for the primal MFP*λ* and let (*u*, *y*, *v*, *w*) be feasible for dual SMMFD*λ*. If

(i) ∑_*i*=1_^*k*^ *λ*_*i*_ *G*_*i*_(·) is second order *ρ*-pseudounivex with respect to *η*, *ψ*_0_, *K*, and for *y*_*J*_2__ ∈ *ℝ* ^*m*−|*J*_1_|^, *y*_*J*_2__^*T*^ *h*_*J*_2__(·) is second order *σ*-quasiunivex with respect to *η*, *ψ*_1_, and *K* along with
(ii) *ρ* + *σ* ≥ 0, then Inf⁡⁡(*λF*(*x*)) ≥ *Sup*⁡(*λG*(*u*)).

**Proof**

Now from the primal and dual constraints, we have

(41) $$\begin{array}{c} h\left(x\right)\leq 0,\\ y_{J_{2}}^{T}h_{J_{2}}\left(u\right)-\frac{1}{2}p^{T}\nabla^{2}\left(y_{J_{2}}^{T}h_{J_{2}}\left(u\right)\right)p\geq 0. \end{array}$$

So

(42) yJ2ThJ2(x)−yJ2ThJ2(u)+12pT∇2(yJ2ThJ2(u))p≤0 ⟹K(x,u)ψ1[12yJ2ThJ2(x)−yJ2ThJ2(u)ssssssssssssssssssss+12pT∇2(yJ2ThJ2(u))p]≤0.

Since *y*_*J*_2__^*T*^ *h*_*J*_2__ is second order *σ*-quasiunivex with respect to *η*,  *ψ*_1_, and *K* and in view of (42), for *x*, *u* ∈ *ℝ* ^*n*^, we have

(43) $$\begin{array}{c} \eta {\left(x,u\right)}^{T}\left\{\nabla \left[y_{J_{2}}^{T}h_{J_{2}}\left(u\right)\right]+\nabla^{2}\left[y_{J_{2}}^{T}h_{J_{2}}\left(u\right)\right]p\right\}+\sigma {||x-u||}^{2}\leq 0. \end{array}$$

Again from the dual constraint (36), we have

(44) ∑i=1kλi[∇Gi(u)+∇2Gi(u)p] +yJ2TT[∇hJ2(u)+∇2hJ2(u)p]=0.

Since *η*(*x*, *u*) ∈ *ℝ* ^*n*^, we have

(45) η(x,u)T{∑i=1kλi[∇Gi(u)+∇2Gi(u)p]}  +η(x,u)T{yJ2T[∇hJ2(u)+∇2hJ2(u)p]}=0, ⟹η(x,u)T{∑i=1kλi[∇Gi(u)+∇2Gi(u)p]}  +η(x,u)T{yJ2T[∇hJ2(u)+∇2hJ2(u)p]}  +σ||x−u||2−σ||x−u||2=0.

Using (43) in above equation, we get *η*(*x*,*u*)^*T*^{∑_*i*=1_^*k*^ *λ*_*i*_[∇*G*_*i*_(*u*) + ∇^2^ *G*_*i*_(*u*)*p*]} − *σ*||*x*−*u*||^2^ ≥ 0.

Since *ρ* + *σ* ≥ 0, we get *ρ*||*x*−*u*||^2^ ≥ −*σ*||*x*−*u*||^2^.

So, we have

(46) $$\begin{array}{c} \eta {\left(x,u\right)}^{T}\left\{\overset{k}{\underset{i=1}{\sum }}\lambda_{i}\left[\nabla G_{i}\left(u\right)+\nabla^{2}G_{i}\left(u\right)p\right]\right\}+\rho {||x-u||}^{2}\geq 0. \end{array}$$

Since ∑_*i*=1_^*k*^ *λ*_*i*_ *G*_*i*_(*u*) is second order *ρ*-pseudounivex with respect to *η*, *ψ*_0_, and *K*, by Definition 2 and (46), we get *K*(*x*, *u*)*ψ*_0_{∑_*i*=1_^*k*^ *λ*_*i*_ *G*_*i*_(*x*) − ∑_*i*=1_^*k*^ *λ*_*i*_ *G*_*i*_(*u*) + (1/2)*p* ^*T*^∑_*i*=1_^*k*^ *λ*_*i*_ *G*_*i*_(*u*)*p*} ≥ 0.

Using the property of *ψ*_0_ and *K*, we get

(47) ∑i=1kλiGi(x)−∑i=1kλiGi(u)+12pT∑i=1kλiGi(u)p≥0 ⟹∑i=1kλiGi(x)≥∑i=1kλiGi(u) ⟹∑i=1kλi[fi(x)+yJ1ThJ1(x)ssssssszzssss+xTBiw−vi{gi(x)−xTCiz}] ≥∑i=1kλi[fi(u)+yJ1ThJ1(u)sssssssssss+uTBiw−vi{gi(u)−xTCiz}].

Equation (5) gives *h*(*x*) ≤ 0⇒*y*_*J*_1__^*T*^ *h*_*J*_1__(*x*) ≤ 0, for *y*_*J*_1__ ≥ 0.

So (47) implies that

(48) ∑i=1kλi[fi(x)+xTBiw−vi{gi(x)−xTCiz}] ≥∑i=1kλi[fi(u)+yJ1ThJ1(u)+uTBiwssssssssssss−yJ1ThJ1vi{gi(u)−uTCiz}].

Now by Schwarz Inequality and (39), we have

(49) xTBiw≤(xTBix)1/2(wTBiw)1/2≤(xTBix)1/2,xTCiz≤(xTCix)1/2(zTCiz)1/2≤(xTCix)1/2,hhhhhhhhhhhhhhhhhhhhhhhhi=1,2,…,k.

So both (48) and (49) imply that

(50) ∑i=1kλi[fi(x)+(xTBix)1/2−vi{gi(x)−(xTCix)1/2}] ≥∑i=1kλi[fi(u)+yJ1ThJ1(u)+uTBiwssssssssssss−vi{gi(u)−uTCiz}] ⟹Inf⁡(λF(x))≥Sup⁡(λF(u)).

**Theorem 14 (strong duality).**

Let $\overset{-}{x}$ be optimal solution for MFP*λ* and let $W\left(\overset{-}{x}\right)=\phi$. Then there exist *λ*_*i*_ ∈ *ℝ*_+_; *w*, *z* ∈ *ℝ* ^*n*^, *v*_*i*_ ∈ *ℝ*_+_, and *y* ∈ *ℝ* ^*m*^ such that $\left(\overset{-}{u},\lambda ,y,v,w,z,p=0\right)$ is a feasible solution for dual and the objective values of both primal and dual are equal to zero. Furthermore if (i) $\left(\overset{-}{u},\lambda ,y,v,w,z\right)$ is feasible for dual, (ii) ∑_*i*=1_^*k*^ *λ*_*i*_ *G*_*i*_(·) is second order *ρ*-pseudounivex with respect to *η*,  *ψ*_0_, and *K* and for *y*_*J*_2__ ∈ *ℝ* ^*m*−|*J*_1_|^, *y*_*J*_1__^*T*^ *h*_*J*_2__(·) is second order *σ*-quasiunivex with respect to *η*,  *ψ*_1_, and *K* along with (iii) *ρ* + *σ* ≥ 0, then $\left(\overset{-}{u},\lambda ,y,v,w,z,p=0\right)$ is properly efficient for SMMFD0.

**Proof**

Since $\overset{-}{x}$ is optimal solution for (MFP*λ*), by Lemma 10, there exist *λ*_*i*_ ∈ *ℝ*_+_; *w*, *z* ∈ *ℝ* ^*n*^, *v*_*i*_ ∈ *ℝ*_+_, and *y* ∈ *ℝ* ^*m*^ such that

(51) ∑i=1kλi[∇fi(x−)+Biw−vi{∇gi(x−)−Ciz}]+yT∇h(x−)=0,fi(x−)+(x−TBix−)1/2−vi(gi(x−)−(x−TCix−)1/2)=0,hhhhhhhhhhhhhhhhhhhhhhhhhhhhi=1,2,…,k,yTh(x−)=0,wTBiw≤1, zTCiz≤1, i=1,2,…,k,(x−TBix−)1/2=x−TBiw,  (x−TCix−)1/2=x−TCiz,hhhhhhhhhhhhhhhhhhhhhhhhhi=1,2,…,k,y≥0,vi≥0, i=1,2,…,k,

which can be written as

(52) ∑i=1kλi[∇fi(x−)+yJ1T∇hJ1(x−)+Biw−vi{∇gi(x−)−Ciz}] +yJ1T∇hJ2(x−)=0,fi(x−)+(x−TBix−)1/2−vi{gi(x−)−(x−TCix−)1/2} +yJ1ThJ1(x−)=0,yJ1ThJ2(x−)=0,wTBiw≤1, zTCiz≤1, i=1,2,…,k,(x−TBix−)1/2=x−TBiw,  (x−TCix−)1/2=x−TCiz,hhhhhhhhhhhhhhhhhhhhhhhhhi=1,2,…,k,y≥0,vi≥0, i=1,2,…,k.

These are nothing but the dual constraints.

So $\left(\overset{-}{u},\lambda ,y,v,w,z,p=0\right)$ is feasible solution for dual problem.

And the objective values of MFP*λ* and SMMFD*λ* are equal to zero. It follows from Theorem 13 and for any feasible solution $\left(\overset{-}{u},\lambda ,y,v,w,z,p=0\right)$ of dual that $\lambda G\left(\overset{-}{u}\right)\leq \lambda G\left(\overset{-}{x}\right)$.

So $\left(\overset{-}{u},\lambda ,y,v,w,z,p=0\right)$ is optimal solution of SMMFD*λ*. Then applying Lemmas 8 and 9, we conclude that $\left(\overset{-}{u},\lambda ,y,v,w,z,p=0\right)$ is properly efficient for (SMMFD0).

## 4. Special Case

If *p* = 0, *C*_*i*_ = 0, *i* = 1,2,…, *k*, then our dual programming reduces to the dual programming proposed by Tripathy [14].

## 5. Conclusion

In this paper, three approaches given by Dinklebaeh [28] and Jagannathan [12] for both primal and second order mixed type dual of a nondifferentiable multiobjective fractional programming problem are introduced and the necessary and sufficient optimality conditions are established and a parameterization technique is used to establish duality results under generalized second order *ρ*-univexity assumption. The results developed in this paper can be further extended to higher order mixed type fractional problem containing square root term. Also the present work can be further extended to a class of nondifferentiable minimax mixed fractional programming problems.
